# Surgical Treatment of Mandibular Coronoid Process Hypertrophy Syndrome

**DOI:** 10.3390/jcm14144931

**Published:** 2025-07-11

**Authors:** Julia Miaśkiewicz, Anna Lato, Michał Gontarz, Krzysztof Gąsiorowski, Tomasz Marecik, Grażyna Wyszyńska-Pawelec, Jakub Bargiel

**Affiliations:** Department of Cranio-Maxillo-Facial Surgery, Oncology and Reconstructive Surgery, Jagiellonian University Medical College, 31-501 Kraków, Poland; julia.miaskiewicz@student.uj.edu.pl (J.M.); anna.lato@student.uj.edu.pl (A.L.); michal.gontarz@uj.edu.pl (M.G.); krzysztof.gasiorowski@uj.edu.pl (K.G.); tomasz.marecik@uj.edu.pl (T.M.); grazyna.wyszynska-pawelec@uj.edu.pl (G.W.-P.)

**Keywords:** mandibular coronoid process, coronoidectomy, intraoral surgical approach, mandibular hypertrophy, oral surgery, limited mouth opening

## Abstract

**Background/Objectives**: Mandibular coronoid process hypertrophy (MCPH) is a rare condition characterized by an abnormal enlargement of the mandibular coronoid process, resulting in restricted mouth opening and reduced lateral mandibular movements due to interference with the zygomatic bone. The objective of this paper is to evaluate the functional outcomes of intraoral coronoidectomy followed by physiotherapy in five consecutive patients with MCPH. **Methods**: Five male patients (mean age 38 ± 18.7 years) with radiologically confirmed bilateral MCPH underwent intraoral coronoidectomy between May 2020 and December 2022. Maximal inter-incisal opening (MIO) was measured pre-operatively, on postoperative day 1, and at 6-month follow-up. A standardized 5-10-60 mouth-opening exercise protocol using a Heister mouth gag was administered from postoperative day 1. **Results**: The hyperplastic mandibular coronoid processes were removed intraorally without any long-lasting complications. All patients demonstrated a postoperative increase in mouth opening. Notably, patients with more severe mouth-opening limitations showed greater improvement compared with those with milder initial restrictions. **Conclusions:** Intraoral coronoidectomy, combined with early and intensive physiotherapy, represents a safe and effective treatment for MCPH. Early diagnosis and timely surgical intervention are crucial to prevent ineffective non-surgical management. Additionally, a modest initial postoperative increase in mouth opening should not be considered the final outcome, as these patients often achieve substantial long-term functional improvement.

## 1. Introduction

The mandibular coronoid process is a triangular, hook-shaped or round bony projection that serves as the attachment for the temporalis muscle [1]. Although the coronoid process is not part of the temporomandibular joint, its elongation can affect mandibular movements [2]. Mandibular coronoid process hypertrophy (MCPH) is a rare condition defined by excessive development of the mandibular coronoid process with normal histology and mainly affects adolescent males, being bilateral in approximately 80% of cases [3]. This leads to progressive impingement of the coronoid process on the body of the zygomatic bone, resulting in reduced mouth opening and lateral movements of the mandible. MCPH was initially described by Langenbeck in 1853, with the first documented case of mandibular hypomobility due to coronoid process enlargement reported in 1899 [4,5].

Radiologic confirmation of MCHP relies on panoramic radiography (OPG) or cone-beam computed tomography (CBCT). It may also be detected incidentally by computed tomography (CT) or magnetic resonance imaging (MRI) obtained for unrelated indications. The condition is confirmed when the height of the mandibular condylar process extends more than 1 cm above the inferior border of the zygomatic arch [6,7]. Treatment is recommended when the elongated coronoid process limits mouth opening or lateral movements of the mandible.

The primary treatment modality for MCHP involves the surgical removal of the elongated process, either by complete resection (coronoidectomy) or detachment of the coronoid process from the ramus (coronoidotomy). These procedures may be performed by intraoral or external approaches [8,9]. There is currently no consensus regarding which technique is superior. Intraoral approach eliminates scar of the skin and reduces the risk of facial nerve injury but enhances internal scarring [10]. Although non-surgical approaches, such as physiotherapy and occlusal splints, have been attempted, these methods generally provide limited improvement. It is thought that only with early diagnosis it is possible to manage the condition without surgery by closely monitoring growth until adulthood, often with good results. However, there are not many studies that examine different treatment options or track patients long-term to assess the durability of those results [11].

The present study describes a homogeneous series of five patients with MCPH managed by intraoral coronoidectomy and a unified physiotherapy protocol, with an emphasis on functional outcomes and the practical aspects of perioperative care.

## 2. Materials and Methods

### 2.1. Patient Selection

In this retrospective case series, we reviewed the medical records of five patients with MCHP treated in the Department of Cranio-Maxillofacial Surgery, Jagiellonian University Hospital in Krakow, Poland, conducted between May 2020 and December 2022.

The inclusion criteria were patients with a confirmed diagnosis of MCPH based on imaging findings and the mandibular condylar process height extending more than 1 cm above the inferior border of the zygomatic arch, age of 18 years or older, and reported difficulties in consuming larger or solid foods. Patients were excluded if the limitation of mouth opening was caused by other etiologies, if an elongated coronoid process was present without functional impairment, if there was evidence of temporomandibular joint ankylosis or other intra-articular pathology, or if the patient did not consent to surgical intervention. All included patients underwent intraoral coronoidectomy under general anesthesia. The surgical approach and procedure duration were recorded for each case. Although no formal comparator group was included, outcomes were evaluated in comparison to preoperative baseline values. The collected data included demographic information, medical history, radiological findings, symptom duration, preoperative maximum interincisal opening (MIO), and the presence of audible painless clicking sounds associated with friction between the hypertrophic coronoid process and the zygomatic bone. Postoperative outcomes and follow-up data were also collected. Mouth opening was assessed using the TheraBite Range of Motion Scale during follow-up evaluations.

### 2.2. Imaging

**Figure 1:**
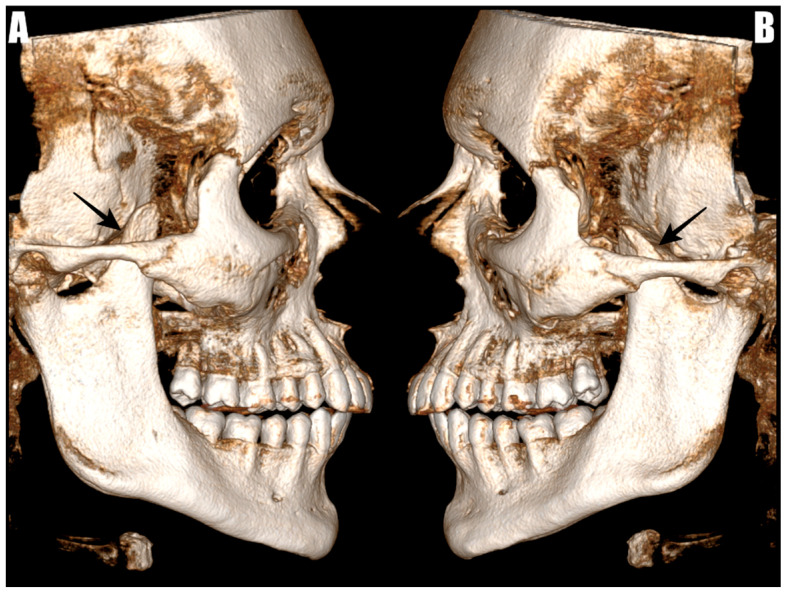
The 3D reconstruction of a CBCT image showing elongated coronoid processes indicated by arrows. (**A**) Represents the right side and (**B**) represents the left side.

Before admission, all patients underwent CBCT using the CS 9600 system (Carestream Dental, Atlanta, GA, USA). The coronoid processes were evaluated through the RadiAnt DICOM Viewer software version 2022.1.1, accessed from https://www.radiantviewer.com (accessed on 17 August 2022). The measurements took place in multi-planar reconstructions (MPRs) and 3D reconstructions. Radiological imaging of elongated coronoid processes with their superior positioning in relation to the zygomatic arch are shown in (Figure 1).

### 2.3. Statystical Analysis

All data was collected and retrospectively reviewed. Continuous variables were presented as mean ± standard deviation (SD). Normality of distributions was assessed using the Shapiro–Wilk test. Categorical variables were reported as counts and percentages. Predictive analyses for postoperative outcomes were performed using univariate logistic regression when the outcome was categorical (e.g., presence of complications), and univariate linear regression when the outcome was continuous (e.g., postoperative maximum mouth opening). Multivariate regression was not performed due to the limited number of outcome events. All analyses were performed using PS IMAGO PRO 10 (SPSS). A two-sided *p*-value < 0.05 was considered statistically significant.

### 2.4. Surgical Technique

**Figure 2:**
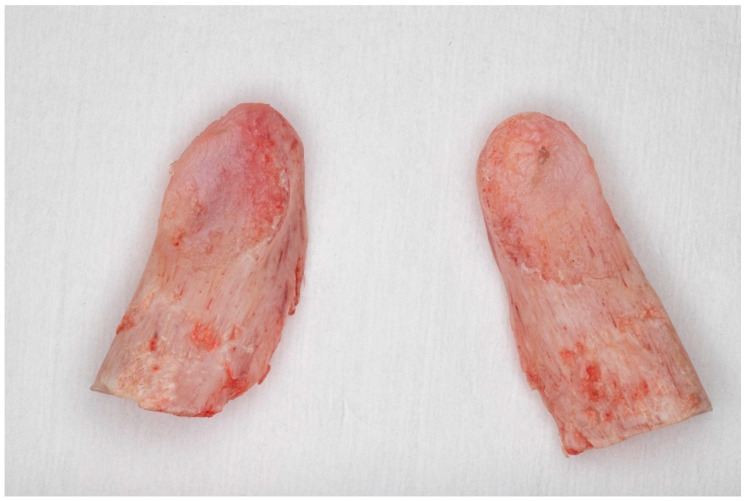
Removed coronoid processes.

The procedure was performed under general anesthesia with nasotracheal intubation and the administration of muscle relaxants. A standard intraoral incision was made approximately 5 mm below the attached gingiva in the second premolar region and extended along the external oblique ridge of the mandible. A subperiosteal dissection was then carried out, exposing both the lateral and medial aspects of the mandibular ramus above the mandibular lingula. The temporalis muscle attachment to the coronoid process was partially released using a Colorado microdissection needle. Two curved Molt elevators were used to identify the mandibular notch, ensuring a clear surgical pathway. Long straight Kocher forceps were then introduced perpendicularly to the occlusal plane and positioned at the base of the coronoid process. The Lindemann burr was then used to carefully section the coronoid process along a safe trajectory aligned with the forceps, extending to the level of the mandibular notch. After mobilization, the coronoid process was gently rotated back and forth to facilitate the release of any residual soft tissue attachments before its safe removal (Figure 2). These residual attachments were subsequently cauterized and divided with scissors. The surgical site was closed in layers using resorbable sutures. Antibiotic therapy consisting of cefuroxime or clindamycin was administered for 5 to 7 days.

### 2.5. Physical Therapy

Physical therapy protocols were initiated on the first day following surgery to facilitate connective tissue stretching, mobilize the temporomandibular joints, and enhance muscle function. Rehabilitation included mouth-opening exercises utilizing a Heister mouth gag. Patients were instructed to place the mouth-opening device between their upper and lower premolars or molars and to gradually unscrew it, stretching the masticatory muscles within a tolerable level of discomfort. As part of the 5-10-60 regimen, patients performed five sessions daily, each consisting of ten openings and closings, with a sixty-second stretch dedicated to each opening. This routine was maintained throughout the first two weeks of rehabilitation. During the third and fourth weeks, the frequency of daily sessions was gradually reduced to three, tapering to one session per day by the sixth or seventh week. Patients were advised to maintain their exercise regimen, with follow-up evaluations planned at three months to monitor progress, and a final assessment of treatment outcomes conducted at six months.

## 3. Case Presentation

### 3.1. Case

A 20-year-old male presented with a painless clicking sound on both sides during the final stage of mouth opening. Intraoral palpation revealed bilateral impingement of the coronoid processes against the zygomatic bones. Imaging confirmed bilateral coronoid hypertrophy.

Preoperative maximal mouth opening (MMO) was 25 mm, increasing to 35 mm on the first postoperative day. At the six-month follow-up, MMO reached 42 mm.

### 3.2. Case 2

A 30-year-old male presented with a significant limitation in mouth opening, with an interincisal distance of 20 mm and painless bilateral clicking during mandibular movement. The radiological evaluation confirmed bilateral coronoid hypertrophy.

On the first postoperative day, the maximum mouth opening (MMO) increased to 30 mm. At the six-month follow-up, MMO reached 38 mm.

### 3.3. Case 3

A 52-year-old patient reported a severe limitation in mouth opening (15 mm) with associated bilateral clicking sounds. The symptoms had been present for over 20 years, without pain or history of trauma. Computed tomography revealed bilateral enlargement of the coronoid processes.

Postoperatively, MMO increased to 30 mm on the first day and to 44 mm at six months.

### 3.4. Case 4

A 64-year-old patient presented with long-standing painless restriction in mouth opening, reportedly progressing since early childhood and without other associated symptoms. Radiological evaluation confirmed bilateral enlargement of the coronoid processes.

Preoperative MMO was 15 mm, increasing to 20 mm on the first postoperative day. At the six-month follow-up, MMO improved to 30 mm. Postoperative complications included mild trismus and a localized hematoma, both managed conservatively.

### 3.5. Case 5

A 26-year-old patient presented with a painless limitation in mouth opening measured at 22 mm, persisting for approximately four years and without other associated symptoms. Radiological evaluation confirmed bilateral coronoid hypertrophy.

MMO increased to 28 mm on the first postoperative day and to 46 mm at the six-month follow-up.

A summary of patient characteristics and treatment details is provided in Table 1.

## 4. Results

Between May 2020 and December 2022, five patients (aged 38 ± 18.7 years, 100% male) met the inclusion criteria and were admitted for resection of an elongated coronoid process.

Preoperative symptoms included 60% (*n* = 3) cases of an audible painless clicking sound during the final stage of mouth opening. In the three cases where presence preoperative clicking was reported, the sound did not originate from the temporomandibular joint (TMJ). As described, it was a painless extra-articular clicking caused by friction between the hypertrophic coronoid process and the zygomatic bone during jaw movement. The clicking resolved completely after coronoidectomy, confirming its non-articular origin.

**Figure 3:**
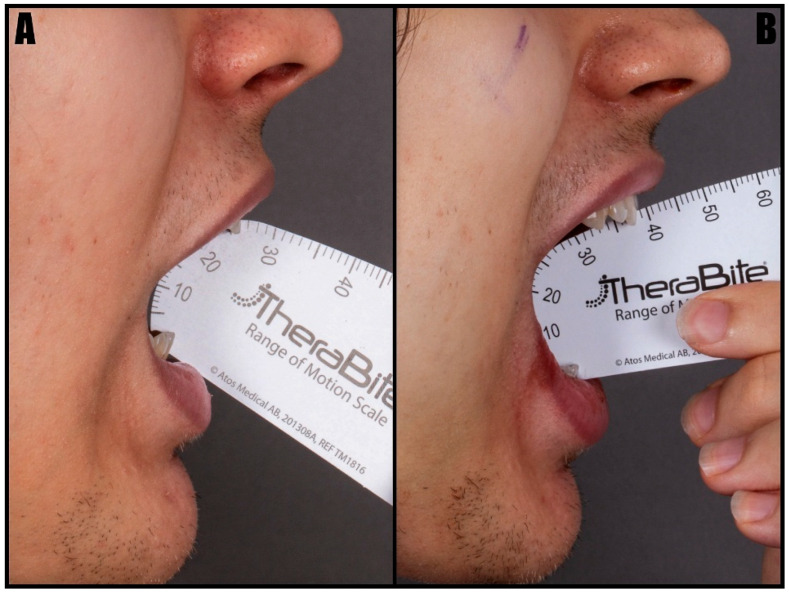
The patient’s mouth opening before (**A**) and on day one (**B**) after the intraoral resection of the elongated coronoid processes.

All patients experienced an improvement in maximum mouth opening following surgery. The initial surgical outcome was enhanced by postoperative physiotherapy and further consolidated after complete healing, which was assessed six months postoperatively. The first postoperative increase averaged 9.2 ± 4 mm. After six months, with the addition of physiotherapy, the mean improvement in maximum mouth opening reached 11.4 ± 4.6 mm. Patient 1’s enhancement in maximum mouth opening postoperatively and on day one after the operation is presented below (Figure 3).

**Figure 4:**
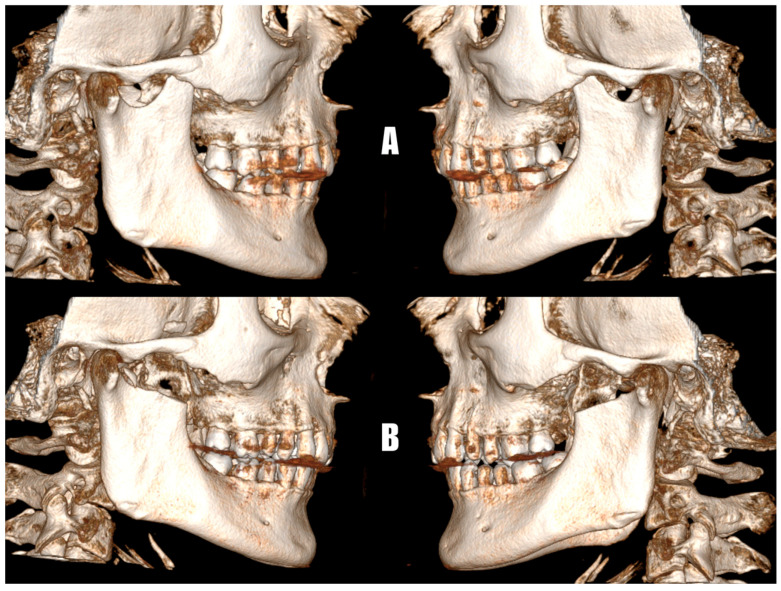
Three-dimensional reconstruction of the patient’s CBCT scans showing the coronoid processes before (**A**) and after (**B**) surgical resection.

All resections of the elongated coronoid processes were performed using an intraoral approach. The average operative time was 64 ± 11.4 min. No significant complications were observed except for transient trismus in three patients, one of whom also developed a hematoma treated with drainage through the wound. In conducted analysis, no statistically significant predictors of postoperative complications were identified in our study. Univariate logistic and linear regression analyses found no significant associations between variables like age and preoperative maximum mouth opening and postoperative outcomes, including postoperative maximum mouth opening and the presence of complications (*p*-value > 0.05). Within this group, these variables seem to have little effect on the risk of complications or postoperative maximum mouth opening, however, larger studies are necessary to confirm these results. To illustrate the surgical outcome following coronoid process resection, a comparison of preoperative and postoperative CBCT images from one representative patient is presented below (Figure 4).

Outcomes following surgical intervention are demonstrated in Table 2.

## 5. Discussion

Mandibular coronoid process hypertrophy (MCPH) is an uncommon condition that is often underdiagnosed, primarily because it progresses slowly and is typically asymptomatic [12]. Although significant restrictions in mouth opening are frequently present from childhood or adolescence, the lack of pain often contributes to delayed diagnosis [13]. Therefore, a comprehensive understanding of its clinical manifestations, along with the appropriate use of diagnostic imaging, is essential for early detection and effective management.

Limited or restricted mouth opening (LMO), also known as chronic mandibular hypomobility, is a common concern in maxillofacial surgical practice. In healthy individuals, maximal interincisal opening typically ranges from 30 to 50 mm, whereas an opening of 20 mm or less indicates pathology. Males generally exhibit greater mouth opening compared with females [14]. Normal lateral jaw movement is 8–12 mm, while protrusive movement is approximately 10 mm [15]. In our cohort, maximum mouth opening did not exceed 25 mm. Although coronoidectomy was successfully performed in all cases, the postoperative maximal mouth opening (MMO) remained below 45 mm in most patients. This finding is clinically significant, as the average mouth opening in healthy adult Polish males typically exceeds 45 mm [16,17]. In patients with late-diagnosed MCHP, restricted mouth opening appears to result from secondary myofascial soft-tissue alterations, which are further influenced by scar formation that develops during healing and subsequent physiotherapy [18,19]. Although intraoperative assessment often reveals notable improvements in mouth opening following coronoidectomy, these findings do not conclusively indicate that the preoperative limitation was solely of a mechanical or osseous origin [20]. Notably, even when the maximal interincisal distance remains below the reference value for the general population, patients report significant improvements in quality of life, particularly with respect to their ability to chew and ingest solid foods, as was the case with our patients. Due to the often prolonged course of the disease and the lack of baseline data regarding mandibular mobility prior to symptom onset, comparisons with normative values are limited.

LMO may result from various underlying conditions and requires a thorough differential diagnosis. Mandibular coronoid process hypertrophy is one of the rarest causes of limited mouth opening, accounting for roughly 5% of cases [21]. While this condition typically exhibits specific clinical features, it can easily be misdiagnosed as temporomandibular joint disorder (TMJD) before radiographic evaluation [22]. Radiographic imaging is necessary to identify the enlarged coronoid process and its relation to adjacent structures, particularly the zygomatic bone. Cone-beam computed tomography is the recommended imaging modality for accurate assessment.

Other causes of LMO include odontogenic infections, such as abscesses, pericoronitis, and osteomyelitis, which account for 24% of cases [23,24,25]. Trismus should be regarded as an oncologic red-flag symptom. When it presents acutely, it may represent the first clinical manifestation of an advanced head-and-neck malignancy [26,27]. In addition, trismus frequently emerges as a late effect of radiotherapy. Post-radiotherapy fibrosis typically develops within six months of irradiation and is reported to affect approximately 10–40% patients [28,29]. Additionally, post-surgical scarring can contribute to LMO due to fibrosis and adhesions, potentially leading to fibrous or extra-articular ankylosis [30]. Effective postoperative rehabilitation, including physiotherapy and jaw exercises, is crucial for reducing the impact of scarring on mouth opening [31].

Coronoid process hypertrophy is a rare disease more frequently observed in males, with a reported male-to-female ratio of 5:1 [10]. This finding is consistent with our case series, which comprised exclusively male patients. Owing to the rarity of this condition, comprehensive epidemiological studies are lacking. Typically, MCHP manifests during the second decade of life [32]. The exact etiology of MCHP remains uncertain, though several hypotheses have been proposed. These include genetic inheritance, hormonal stimulation, facial injuries or trauma, and increased temporal muscle activity [33].

MCPH can significantly impact an individual’s psychological well-being and social life. In our cohort, all patients reported subjective improvement in quality of life after the procedure. Specifically, they expressed satisfaction with the treatment outcomes and reported the ability to consume all types of food without difficulty. Moreover, they experienced significant improvement in mandibular function, including unrestricted mouth opening and absence of mechanical obstruction during mastication or speech. Additionally, it can affect a patient’s eating habits, making it difficult to consume certain foods that demand extensive chewing [34]. In all our cases, three patients have reported experiencing long-term crackle sounds on the site of coronoid process hypertrophy. Furthermore, they indicated difficulties consuming larger portions of solid foods without prior segmentation into smaller portions.

The method of choice for restoring mouth opening in MCPH is surgical removal followed by physiotherapy [33]. Surgical treatment can be performed through either intraoral or extraoral access and may involve complete resection (coronoidectomy) or detachment of the coronoid process from the ramus (coronoidotomy). Regarding our procedures, we implemented an intraoral approach in coronoidectomy. The extraoral approach is especially valuable when the coronoid process is significantly elongated [35]. It offers advantages in terms of visibility compared with the intraoral approach. Nevertheless, it is associated with potential aesthetic complications, notably visible scarring and an increased risk of facial nerve injury [36]. Additionally, regarding the extraoral approach, there is a risk of sensory disturbances in the forehead and parietal area [37]. In most cases, intraoral coronoidectomy is the preferred option, as it results in a shorter recovery period compared with a more invasive extraoral approach. However, in some situations, the intraoral approach may limit clear visualization of the osteotomy line [38]. The most common complications are postoperative fibrosis and hematoma [39]. To prevent such complications, the endoscopic-assisted intraoral approach is considered effective [40]. Besides its limited role in jaw function, the coronoid process can be safely removed without causing functional problems [41]. In our patient cohort, no significant complications were observed other than transient postoperative trismus in three patients. The excised coronoid process may also be an effective bone grafting material, particularly for reconstructive procedures involving the orbital floor or temporomandibular joint, as indicated in previous studies [40,41,42].

Recent findings suggest that the early initiation of physiotherapy, such as within one or two weeks after intraoral surgery, improves mouth opening [43,44]. Accordingly, physiotherapy was commenced on the first postoperative day in our protocol. The key factor behind physiotherapy’s success in treatment is its consistency [45,46,47]. Our patients were instructed to follow the 5-10-60 regime. The mentioned regime is based on five sessions per day with ten openings and closing per session and sixty seconds stretch for each opening. Active jaw exercises proved to be effective in the management of restricted mouth opening [48]. Mouth gags were primarily designed to keep the mouth open during surgical procedures in the mouth and throat regions [49]. Additionally, they were used by anesthesiologists to provide better access to the upper airway [50]. Recently, however, they have been incorporated into postoperative physiotherapy routines that patients can safely use at home [51]. Moreover, the same regime and frequency can be implemented with the use of mouth opening exercise devices, such as Therabite, Dynasplint, and EZbite [52,53]. These devices are typically simple hand-operated instruments that employ a wedge or lever mechanism [54]. Patients are asked to place the device between the upper and lower teeth and modify to gently stretch the jaw muscles. As with passive mouth-opening devices such as the spatula method, the patient is instructed to note the level of retraction using the device’s scale on their first day of mechanotherapy.

Non-surgical approaches, including prolonged physiotherapy, occlusal splints, and temporizing muscle relaxation techniques, have been explored—particularly in pediatric or high-risk patients. However, these methods generally provide only limited and often temporary functional improvement [11]. Long-term outcomes of conservative treatment remain variable and are highly dependent on patient compliance. In most cases, mechanical obstruction caused by the hypertrophic coronoid process persists, rendering non-surgical approaches largely supportive rather than curative [10]. Nevertheless, non-surgical treatment offers several advantages, such as being non-invasive, avoiding surgical risks and complications, and allowing for initial functional improvement without the need for anesthesia. Additionally, it may serve as a valuable option for patients who are not candidates for surgery due to medical comorbidities or who prefer to delay surgical intervention. Intraoral coronoidectomy has demonstrated significant improvement in maximal mouth opening, yielding good outcomes with minimal complications. Proper clinical assessment, combined with imaging techniques, such as X-ray, CT, or MRI, is crucial for accurate diagnosis and targeted treatment.

Our results should be considered in light of several limitations. As an uncontrolled case series, the absence of a comparison group restricts the capacity to establish causal relationships. Additionally, although typical for this type of study, the relatively small sample size limits the ability to identify rare side effects or subtle variations among subgroups. Moreover, all participants in our study were male, which limits the ability to examine gender-related differences and may affect the generalizability of the findings to both men and women.

## 6. Conclusions

Increasing awareness among healthcare professionals that mandibular coronoid process hypertrophy may cause limited mouth opening supports timely diagnosis and the selection of appropriate treatment, which is essential for reducing patient discomfort. Surgical procedures such as coronoidectomy or coronoidotomy, preferably performed using an intraoral approach, followed by physiotherapy, remain the only effective options for managing this condition. Notably, limited improvement in immediate postoperative mouth opening does not necessarily predict the final outcome, as substantial gains have been observed over time with consistent physiotherapy in the presented cases. Providing appropriate treatment is crucial, as symptoms may persist for years and progressively impair the patient’s quality of life.

## Figures and Tables

**Table 1:** Patient characteristics.

Medical Data	Patients				
	Case 1	Case 2	Case 3	Case 4	Case 5
Age [years]	20	30	52	64	26
Gender	male	male	male	male	male
Duration of symptoms [years]	7	15	20	no precise assessment (since childhood)	4
Mouth opening before surgery [mm]	25	20	15	15	22
Audible painless clicking sound during final stage of mouth opening resulting from friction between the hypertrophic coronoid process and the zygomatic bone.	yes	yes	yes	no	no
Eating habits	Difficulties consuming larger solid foods.				

**Table 2:** Outcomes following surgical intervention.

Outcomes Following Surgical Intervention	Patients				
Data	Case 1	Case 2	Case 3	Case 4	Case 5
Mouth opening before surgery [mm]	25	20	15	15	22
Mouth opening on postoperative day 1 [mm]	35	30	30	20	28
Mouth opening after 6 months [mm]	42	38	44	30	46
Physical therapy	Hiester mouth gag				
Surgical technique	Intraoral approach				
Operative time [min]	60	60	80	70	50
Antibiotic therapy	clindamycin	clindamycin	cefuroxime	cefuroxime	cefuroxime
Complications	trismus	none	trismus	trismus, hematoma	none

## Data Availability

The original contributions presented in this study are included in the article. Further inquiries can be directed to the corresponding author.

## Abbreviations

The following abbreviations are used in this manuscript:

MCPH	mandibular coronoid process hypertrophy
OSMF	oral submucous fibrosis
MMO	maximal mouth opening
LMO	limited mouth opening
OPG	panoramic radiography
CT	computed tomography
CBCT	cone-beam computed tomography
MRI	magnetic resonance imaging
TMJD	temporomandibular joint disorders

## References

[1] Çorumlu U., Kopuz C., Demir M.T., Pirzirenli M.E. (2016). Bilateral elongated mandibular coronoid process in an Anatolian skull. Anat. Cell Biol..

[2] Zhang Y., Liu F., Shen J., Li X. (2022). Two cases of bilateral coronoid process hyperplasia causing restricted mouth opening. Hua Xi Kou Qiang Yi Xue Za Zhi/West China J. Stomatol..

[3] Shujaat S., Politis C., Van Den Bogaert T., Vueghs P., Smeets M., Verhelst P.J., Grymonprez E., Jacobs R. (2023). Morphological characteristics of coronoid process and revisiting definition of coronoid hyperplasia. Sci. Rep..

[4] Von Langenbeck B. (1861). Angeborene Kleinheit Der Unterkiefer. Langenbeck’s Arch. Surg..

[5] Jacob O. (1899). Une Cause Rare de Constriction Permanente Des Machoires. Bull. Mem. Soc. Anat. Paris.

[6] Galiè M., Consorti G., Tieghi R., Denes S.A., Fainardi E., Schmid J.L., Neuschl M., Clauser L. (2010). Early surgical treatment in unilateral coronoid hyperplasia and facial asymmetry. J. Craniofac. Surg..

[7] Izumi M., Isobe M., Toyama M., Ariji Y., Gotoh M., Naitoh M., Kurita K., Ariji E. (2005). Computed tomographic features of bilateral coronoid process hyperplasia with special emphasis on patients without interference between the process and the zygomatic bone. Oral Surg. Oral Med. Oral Pathol. Oral Radiol. Endod..

[8] Newaskar V., Idrees F., Patel P. (2012). Unilateral coronoid hyperplasia treated by coronoidectomy using a transzygomatic approach. Natl. J. Maxillofac. Surg..

[9] Sanromán J.F., Pons M.C., Gallo J.A., Ferro M.F. (2024). Endoscopically Assisted Intraoral Coronoidectomy for Treatment of Coronoid Hyperplasia. J. Craniofac. Surg..

[10] Parmentier G.I.L., Nys M., Verstraete L., Politis C. (2022). A systematic review of treatment and outcomes in patients with mandibular coronoid process hyperplasia. J. Korean Assoc. Oral Maxillofac. Surg..

[11] D’apuzzo F., Minervini G., Grassia V., Rotolo R.P., Perillo L., Nucci L. (2021). Mandibular coronoid process hypertrophy: Diagnosis and 20-year follow-up with CBCT, MRI and EMG evaluations. Appl. Sci..

[12] Nogueira E.F.D.C., Maranhão C.M.D.C.T., Aguiar P.L., Almeida R.D.A.C., Torres B.C.A., Vasconcellos R.J.D.H. (2021). Treatment of hyperplasia of the coronoid process of the mandible in adults: Analysis of 42 literature reports and illustrative case. RGO-Rev. Gaúcha Odontol..

[13] Ghazizadeh M., Sheikhi M., Salehi M.M., Khaleghi A. (2018). Bilateral coronoid hyperplasia causing painless limitation of mandibular movement. Radiol. Case Rep..

[14] Xu X., Shum M., Ting A., Mei L., Guan G. (2020). Estimation of jaw-opening forces, energy expenditure and jaw-opening patterns in adults. Arch. Oral Biol..

[15] Ashmmam M., Rhab M. (2018). Limited Mouth opening causes and its Treatment modalities among Peoples in Al-khoms, Libya. IOSR J. Dent. Med. Sci. (IOSR-JDMS).

[16] Zawawi K.H., Al-Badawi E.A., Lobo S.L., Melis M., Mehta N.R. (2003). An index for the measurement of normal maximum mouth opening. J. Can. Dent. Assoc..

[17] Loster J.E., Groch M., Ryniewicz W., Osiewicz M.A., Wieczorek A. (2016). Assessment of the range of mandibular movements as related to gender in Polish young adult non-patients. J. Stomatol..

[18] Shrivastav S., Bhola N.D., Kambala R., Jadhav A., Hingnikar P., Patil T. (2020). Efficacy of coronoidotomy as an adjunct to fibrotomy in advanced cases of oral submucous fibrosis: A prospective cross-sectional study. J. Datta Meghe Inst. Med. Sci. Univ..

[19] Gibbons A.J., Abulhoul S. (2007). Use of a Therabite appliance in the management of bilateral mandibular coronoid hyperplasia. Br. J. Oral Maxillofac. Surg..

[20] Gupta H., Tandon P., Kumar D., Sinha V., Gupta S., Mehra H., Singh J. (2014). Role of coronoidectomy in increasing mouth opening. Natl. J. Maxillofac. Surg..

[21] Goh Y.C., Tan C.C., Lim D. (2020). Coronoid hyperplasia: A review. J. Stomatol. Oral Maxillofac. Surg..

[22] Mattei L., Raoul G., Barry F., Ferri J., Nicot R. (2024). Is panoramic radiography adequate for diagnosing coronoid process hyperplasia? A case series. J. Stomatol. Oral Maxillofac. Surg..

[23] Neal T.W., Schlieve T. (2022). Complications of Severe Odontogenic Infections: A Review. Biology.

[24] Obradovic B. (2021). Intraoral management of odontogenic infection associated with severe trismus under local anesthesia. Ann. Ital. Chir..

[25] Fu B., McGowan K., Sun J.H., Batstone M. (2020). Increasing frequency and severity of odontogenic infection requiring hospital admission and surgical management. Br. J. Oral Maxillofac. Surg..

[26] van der Geer S.J., van Rijn P.V., Roodenburg J.L.N., Dijkstra P.U. (2020). Prognostic Factors Associated with a Restricted Mouth Opening (Trismus) in Patients with Head and Neck Cancer: Systematic Review. Head Neck.

[27] Sollecito T.P., Helgeson E.S., Lalla R.V., Treister N.S., Schmidt B.L., Patton L.L., Lin A., Brennan M.T. (2024). Reduced mouth opening in patients with head and neck cancer treated with radiation therapy: An analysis of the Clinical Registry of Dental Outcomes in Head and Neck Cancer Patients (OraRad). Oral Surg. Oral Med. Oral Pathol. Oral Radiol..

[28] Aghajanzadeh S., Karlsson T., Tuomi L., Engström M., Finizia C. (2023). Trismus, health-related quality of life, and trismus-related symptoms up to 5 years post-radiotherapy for head and neck cancer treated between 2007 and 2012. Support. Care Cancer.

[29] Weber C., Dommerich S., Pau H.W., Kramp B. (2010). Limited mouth opening after primary therapy of head and neck cancer. Oral Maxillofac. Surg..

[30] Johnson N.W., Warnakulasuriya S., Gupta P.C., Dimba E., Chindia M., Otoh E.C., Sankaranarayanan R., Califano J., Kowalski L. (2011). Global oral health inequalities in incidence and outcomes for oral cancer: Causes and solutions. Adv. Dent. Res..

[31] Wu H., Zhou Z., Zhang C., Shen S., Liu J., Zhang C. (2021). The Progress of Post-Treatment Restricted Mouth Opening in Oral and Maxillofacial Malignant Tumor Patients. Front. Oral Maxillofac. Med..

[32] Cha J., Chung J.W. (2023). Coronoid Process Hyperplasia: A Rare Case of Restricted Mouth Opening Masquerading as Temporomandibular Disorder. J. Oral Med. Pain.

[33] Erdem S., Erdem S. (2022). Investigation of coronoid process hyperplasia using Levandoski analysis on panoramic radiographs. World J. Radiol..

[34] Yost O., Liverman C.T., English R., Mackey S., Bond E.C. (2020). Temporomandibular Disorders: Priorities for Research and Care.

[35] Roberta C., Pasquale P., Giacomo D.R., Massimo M., Stefano V., Rosa V., Ambrosina M., Luigi C. (2019). Mandibular coronoid process tumor resembling a mandibular condyle: A case report. Oral Maxillofac. Surg. Cases.

[36] Mulder C.H., Kalaykova S.I., Gortzak R.A.T. (2012). Coronoid process hyperplasia: A systematic review of the literature from 1995. Int. J. Oral Maxillofac. Surg..

[37] Prabhu R., Sinha R., Chowdhury S.K., Chattopadhyay P. (2012). Evaluation of facial nerve function following surgical approaches for maxillofacial trauma. Ann. Maxillofac. Surg..

[38] Posnick J.C., Kinard B.E. (2021). Use of a ‘low and short’ medial cut limits sagittal ramus osteotomy interferences. Int. J. Oral Maxillofac. Surg..

[39] Kim S.-M., Lee J.-H., Kim H.-J., Huh J.-K. (2014). Mouth opening limitation caused by coronoid hyperplasia: A report of four cases. J. Korean Assoc. Oral Maxillofac. Surg..

[40] Alagarsamy R., Lal B., Arangaraju R., Roychoudhury A., Srivastava R.K., Barathi A. (2023). Endoscopic-assisted intraoral approach for mandibular condyle fracture management: A systematic review and meta-analysis. Oral Surg. Oral Med. Oral Pathol. Oral Radiol..

[41] Mohanty S., Kohli S., Dabas J., Kumar R.D., Bodh R., Yadav S. (2017). Fate of the Coronoid Process After Coronoidotomy and Its Effect on the Interincisal Opening: A Clinical and Radiologic Assessment. J. Oral Maxillofac. Surg..

[42] Sood R., Froimson J.R., Reid R.R. (2024). Simultaneous Coronoid Bone Grafting in Ballistic Facial Trauma Patients Undergoing Coronoidectomy. J. Craniofac. Surg..

[43] Adachi M., Abe A. (2014). Particulated coronoid process grafts in marginal mandibulectomy. J. Oral Maxillofac. Surg..

[44] Amrani S., Anastassov G.E., Montazem A.H. (2010). Mandibular Ramus/Coronoid Process Grafts in Maxillofacial Reconstructive Surgery. J. Oral Maxillofac. Surg..

[45] Ren W.H., Ao H.W., Lin Q., Xu Z.G., Zhang B. (2013). Efficacy of mouth opening exercises in treating trismus after maxillectomy. Chin. Med. J..

[46] Lee R., Molassiotis A., Rogers S.N., Edwards R.T., Ryder D., Slevin N. (2018). Protocol for the trismus trial-Therabite versus wooden spatula in the amelioration of trismus in patients with head and neck cancer: Randomised pilot study. BMJ Open.

[47] Idáñez-Robles A.M., Obrero-Gaitán E., Lomas-Vega R., Osuna-Pérez M.C., Cortés-Pérez I., Zagalaz-Anula N. (2023). Exercise therapy improves pain and mouth opening in temporomandibular disorders: A systematic review with meta-analysis. Clin. Rehabil..

[48] Lindfors E., Arima T., Baad-Hansen L., Bakke M., De Laat A., Giannakopoulos N., Glaros A., Guimarães A., Johansson A., Le Bell Y. (2019). Jaw Exercises in the Treatment of Temporomandibular Disorders—An International Modified Delphi Study. J. Oral Facial Pain. Headache.

[49] Haridas R.P., Wilkinson D.J. (2012). The Phillips Airway. Anaesth. Intensive Care.

[50] Singla K., Samra T., Jain K. (2022). Heister mouth gag aided endotracheal intubation in patients with maxillofacial trauma: A case report. Int. J. Crit. Illn. Inj. Sci..

[51] Lofters A., Clarkson E., Gags M. (2021). Disadvantages. Oral Maxillofac. Surg. Clin. N. Am..

[52] Montalvo C., Finizia C., Pauli N., Fagerberg-Mohlin B., Andréll P. (2017). Impact of exercise with TheraBite device on trismus and health-related quality of life: A prospective study. Ear Nose Throat J..

[53] Li Y.H., Liu C.C., Chiang T.E., Chen Y.W. (2018). EZBite open-mouth device: A new treatment option for oral submucous fibrosis-related trismus. J. Dent. Sci..

[54] Charters E., Dunn M., Cheng K., Aung V., Mukherjee P., Froggatt C., Dusseldorp J.R., Clark J.R. (2022). Trismus therapy devices: A systematic review. Oral Oncol..

